# Supplementary material for: Causal interplay between lactose intolerance and gut microbiota: a combined bidirectional Mendelian randomization and in vivo validation study
Source: Front Nutr. 2026 Jun 1;13:1803337. doi: 10.3389/fnut.2026.1803337 (PMC13265576; doi:10.3389/fnut.2026.1803337)
Supplement: Supplementary file 1 [file Data_Sheet_1.zip › supplementary materials/Forward/leave one out cross validation/ebi-a-GCST90027509.finngen_R12_E4_LACTONAS.pdf]

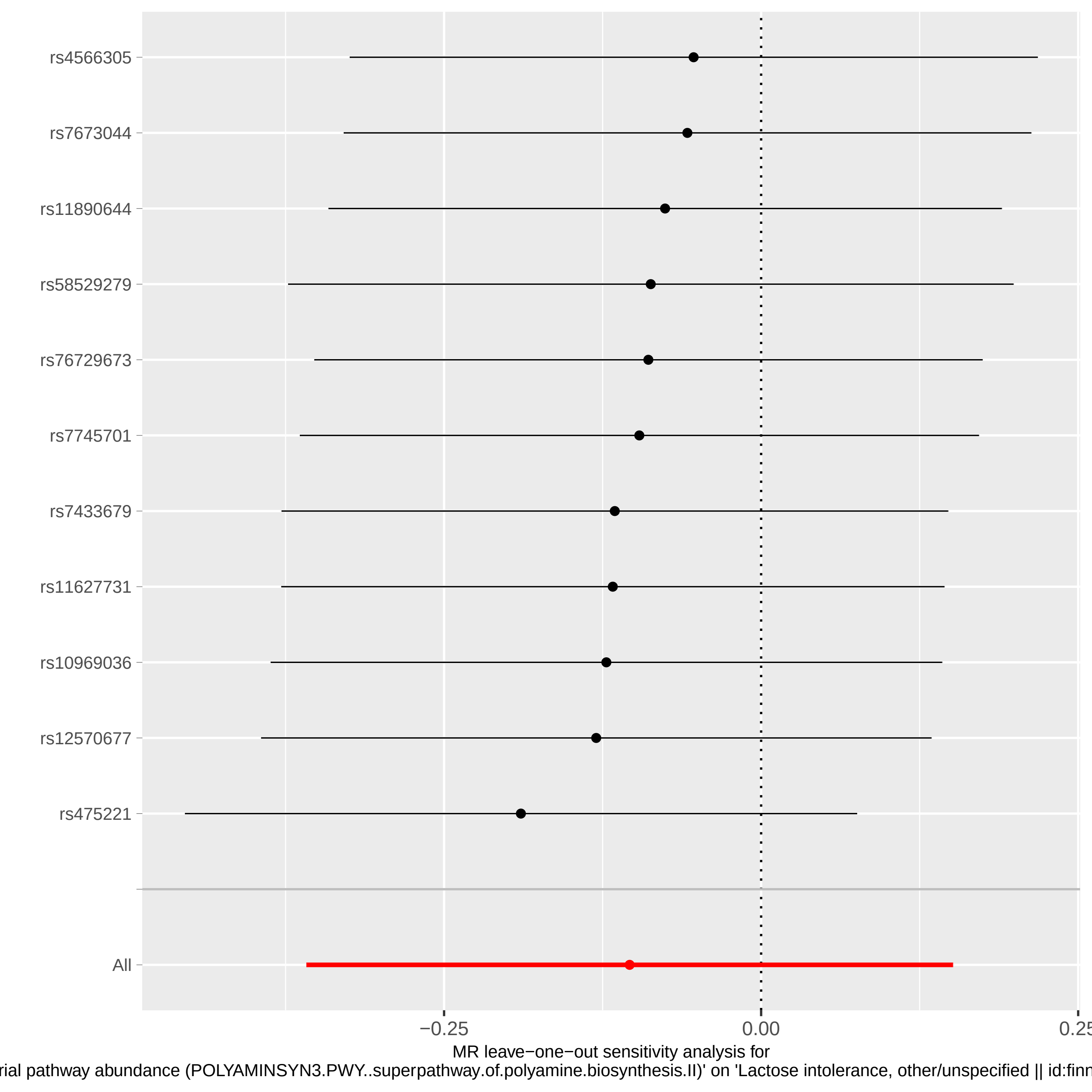
rs4566305
rs7673044
rs11890644
rs58529279
rs76729673
rs7745701
rs7433679
rs11627731
rs10969036
rs12570677
rs475221
All
−0.25
0.00
0.25
MR leave−one−out sensitivity analysis for
rial pathway abundance (POLYAMINSYN3.PWY..superpathway.of.polyamine.biosynthesis.II)' on 'Lactose intolerance, other/unspecified || id:finn
